# Editorial: Insights in disaster and emergency medicine: 2022

**DOI:** 10.3389/fpubh.2023.1212448

**Published:** 2023-06-13

**Authors:** Stefano Orlando

**Affiliations:** Department of Biomedicine and Prevention, University of Tor Vergata, Rome, Italy

**Keywords:** disaster and emergency medicine, climate change, intersectoral collaboration, trauma care, multidisciplinary approach

The year 2022 has been a year of significant advancements in disaster and emergency medicine. The global community, in the previous years, has faced numerous challenges, from the COVID-19 pandemic to an increase in natural disasters (1), and these experiences have provided valuable insights into disaster preparedness, response, and recovery. This Research Topic brings together six outstanding contributions that explore various aspects of disaster and emergency medicine, focusing on public health, military medicine, trauma care, and cardiac arrest management. It aims to shed light on the challenges and advancements in the field of disaster and emergency medicine, emphasizing the importance of a multidisciplinary and collaborative approach (2). The contributing articles in this Research Topic cover a wide range of topics, highlighting the complexity of this field and the need for innovative solutions to address emerging challenges.

Climate change is an essential theme in this Research Topic, as it has far-reaching implications for public health and emergency medicine (3). Robinson et al. explore the impact of climate change on military health and defense medical logistics. The authors identify significant knowledge gaps in understanding the impact of climate change on military health, highlighting the need for further research and investment in military medical research and development. The review points out that climate change may not only affect human physiology and mental health but also have implications for medical logistics, including the cold supply chain, medical devices, air conditioning, and fresh water supply.

Another significant aspect of disaster and emergency medicine is the management of patients in challenging environments. Two articles in this Research Topic address the intricacies of providing medical care during transportation. Post et al. discuss the treatment of intubated patients during aeromedical evacuation flights. Their study of German Air Force's humanitarian aid missions reveals no clinically significant deteriorations due to secondary transport. However, the authors emphasize the importance of elective intubation in borderline patients before flight, considering the hypobaric hypoxic conditions on board aircraft. Borgstedt et al. investigate factors affecting out-of-hospital cardiac arrest outcomes, emphasizing the crucial role of the chain of survival. Their retrospective study of emergency medical service (EMS) protocols in Munich, Germany, reveals that the location of out-of-hospital cardiac arrest (OHCA) does not significantly affect the return of spontaneous circulation incidence, although patients in public spaces have a higher chance of being admitted to the hospital with spontaneous circulation. The study also highlights the overall low levels of bystander CPR and bystander use of automated external defibrillators, emphasizing the importance of public education and training to improve the chain of survival.

The Research Topic also delves into the epidemiology of trauma in China. Wang et al. present a national retrospective study on trauma treatment and incidence, providing valuable data to inform healthcare prevention and management strategies in the country. By analyzing over 4.5 million trauma patients from two national-level databases, the authors offer insights into the demographic characteristics, trauma causes, injury degrees, disease burden, and mortality rates. This valuable information can serve as a foundation for informed decision-making, resource allocation, trauma prevention, and trauma management.

The interconnectedness and multidisciplinary nature of disaster and emergency medicine are further emphasized through the articles exploring the challenges posed by the COVID-19 pandemic. Lamberti-Castronuovo et al. discuss the key role of health diplomacy in overcoming the crisis, demonstrating the importance of intersectoral cooperation and collaboration between different areas of expertise. By conducting a retrospective observational case study with qualitative methodology, the authors analyze the changes made to human resources, health service delivery, and logistics in response to the pandemic. Their findings underscore the importance of intersectoral collaboration in overcoming pandemic-related challenges and preparing for future disasters.

This Research Topic showcases the breadth and depth of disaster and emergency medicine research in 2022, demonstrating the crucial role of intersectoral collaboration, adaptation to changing environments, and effective resource allocation. As we continue to face complex challenges in public health, military medicine, trauma care, and cardiac arrest management, it is essential to draw upon the valuable insights provided by these studies to advance our understanding and enhance our response capabilities.

The diverse range of issues addressed in this Research Topic highlights the interconnectedness and multidisciplinary nature of disaster and emergency medicine. As underscored by Robinson et al., climate change is having and will continue to have a significant impact on public health, particularly in the context of emergency medicine. The increasing frequency and severity of extreme weather events call for the development of adaptive strategies. These strategies should focus on enhancing our capacity to respond to emergencies, especially in resource-limited countries where the consequences of extreme weather events are more severe, and response capabilities are often constrained (4).

Public health, by definition, is multidisciplinary and multisectoral. The contributing articles in this Research Topic demonstrate the indispensable nature of collaboration between different sectors and areas of expertise in emergency disaster response. As highlighted by Lamberti-Castronuovo et al., the COVID-19 pandemic showcased the importance of intersectoral cooperation in overcoming the challenges posed by a global health crisis. However, such collaboration cannot be improvised in the wake of an emergency. Instead, it requires an ongoing relationship between the actors potentially involved in the response, fostering a culture of preparedness and resilience.

In conclusion, this Research Topic underlines the ever-evolving nature of disaster and emergency medicine and the need for collaboration, innovation, and preparedness to address the emerging challenges. The findings and insights from these contributions will undoubtedly contribute to the ongoing efforts to improve the practice of disaster and emergency medicine and enhance our capacity to respond to the pressing issues of our time. As researchers, practitioners, and policymakers continue to work together in this multidisciplinary field, we can expect further advancements that will ultimately benefit the health and wellbeing of communities worldwide.

## Author contributions

SO conceptualized the Research Topic, drafted the manuscript, ensuring that it adhered to the journal guidelines and provided a comprehensive overview of the Research Topic, revised the manuscript based on feedback, ensuring its clarity and coherence, and solely responsible for the content of this editorial and approves its submission to the journal.
